# Expression of Prion Protein in Mouse Erythroid Progenitors and Differentiating Murine Erythroleukemia Cells

**DOI:** 10.1371/journal.pone.0024599

**Published:** 2011-09-02

**Authors:** Martin Panigaj, Hana Glier, Marcela Wildova, Karel Holada

**Affiliations:** Institute of Immunology and Microbiology, First Faculty of Medicine, Charles University in Prague, Prague, Czech Republic; Ohio State University, United States of America

## Abstract

Prion diseases have been observed to deregulate the transcription of erythroid genes, and prion protein knockout mice have demonstrated a diminished response to experimental anemia. To investigate the role of the cellular prion protein (PrP^C^) in erythropoiesis, we studied the protein's expression on mouse erythroid precursors *in vivo* and utilized an *in vitro* model of the erythroid differentiation of murine erythroleukemia cells (MEL) to evaluate the effect of silencing PrP^C^ through RNA interference.

The expression of PrP^C^ and selected differentiation markers was analyzed by quantitative multicolor flow cytometry, western blot analysis and quantitative RT-PCR. The silencing of PrP^C^ expression in MEL cells was achieved by expression of shRNAmir from an integrated retroviral vector genome. The initial upregulation of PrP^C^ expression in differentiating erythroid precursors was detected both *in vivo* and *in vitro*, suggesting PrP^C^'s importance to the early stages of differentiation. The upregulation was highest on early erythroblasts (16200±3700 PrP^C^ / cell) and was followed by the gradual decrease of PrP^C^ level with the precursor's maturation reaching 470±230 PrP^C^ / cell on most mature CD71^−^Ter119^+^ small precursors. Interestingly, the downregulation of PrP^C^ protein with maturation of MEL cells was not accompanied by the decrease of PrP mRNA. The stable expression of anti-Prnp shRNAmir in MEL cells led to the efficient (>80%) silencing of PrP^C^ levels. Cell growth, viability, hemoglobin production and the transcription of selected differentiation markers were not affected by the downregulation of PrP^C^.

In conclusion, the regulation of PrP^C^ expression in differentiating MEL cells mimics the pattern detected on mouse erythroid precursors *in vivo*. Decrease of PrP^C^ protein expression during MEL cell maturation is not regulated on transcriptional level. The efficient silencing of PrP^C^ levels, despite not affecting MEL cell differentiation, enables created MEL lines to be used for studies of PrP^C^ cellular function.

## Introduction

The cellular prion protein (PrP^C^) is expressed in cells of various origins. It is conserved through the whole vertebrae class, suggesting its importance in cellular physiology[1]. However, its role in physiological processes remains enigmatic although PrP^C^ plays a basic role in the pathogenesis of the fatal neurodegenerative disorders known as Transmissible Spongiform Encephalopathies. Observation of PrP^C^ deficient mice (PrP^−/−^) did not reveal significant health problems. On the other hand, experiments in cell cultures suggested that PrP^C^ is linked to such processes as the prevention of apoptosis, copper metabolism linked to oxidative stress, iron metabolism, signalization and differentiation[2], [3], [4]. A connection between prion pathogenesis and erythropoiesis was suggested by the downregulation of the α-hemoglobin stabilizing protein (AHSP) mRNA during prion disease[5]. A later study indicated that the disease progression affected the transcription of several other murine erythroid genes, e.g., Kell, GPA, band 3 and ankyrin[6]. A link between PrP^C^ expression and erythropoiesis was also demonstrated in PrP^−/−^ mice after the experimental induction of hemolytic anemia. Upon treatment with phenylhydrazine, PrP^−/−^ mice produced fewer reticulocytes than did PrP^+/+^ mice. This result was probably due to the reduced level of erythropoietin production and higher percentage of apoptotic erythroid precursors[7]. Apparently, the expression of PrP^C^ is generally important in hematopoiesis. PrP^C^ is present on the surface of mouse long-term hematopoietic stem cells (LT-HSCs) and supports their self-renewal and engraftment during serial transplantation. Quantitatively, 40% of mouse bone marrow (BM) cells express PrP^C^, and, from this population of cells, 80% are erythroid cells[8]. CD34^+^ human bone marrow stem cells also express PrP^C^ [9], [10], and circulating human and mouse red blood cells have similarly low levels of PrP^C^ on their cellular membranes[11], [12]. A widely used model for the study of erythroid differentiation *in vitro* is presented by murine erythroleukemia (MEL) cells. MEL cells are blocked at the proliferative proerythroblast stage, and, after the addition of polar substances, e.g., hexamethylene bisacetamide (HMBA), they lose their proliferative capacity and enter cell-cycle arrest. This process is characterized by structural (decreased cell volume and nuclear condensation) and biochemical changes (activation of erythroid genes, hemoglobin accumulation), which resemble those exhibited by natural erythroid differentiation[13]. Gougoumas and colleagues demonstrated transcriptional activation at the mRNA level of the PrP gene in growth-arrested MEL cells[14]. Our study extends their observations by demonstrating divergences in the regulation of PrP^C^ at the protein and mRNA levels during inducer-mediated erythroid differentiation and cell-growth arrest caused by confluency. In addition, we exploited MEL cell lines with stably downregulated levels of PrP^C^ to study its importance in the differentiation of MEL cells.

## Results

### Regulation of PrP^C^expression on mouse bone marrow and spleen erythroid precursors

**Figure 1:**
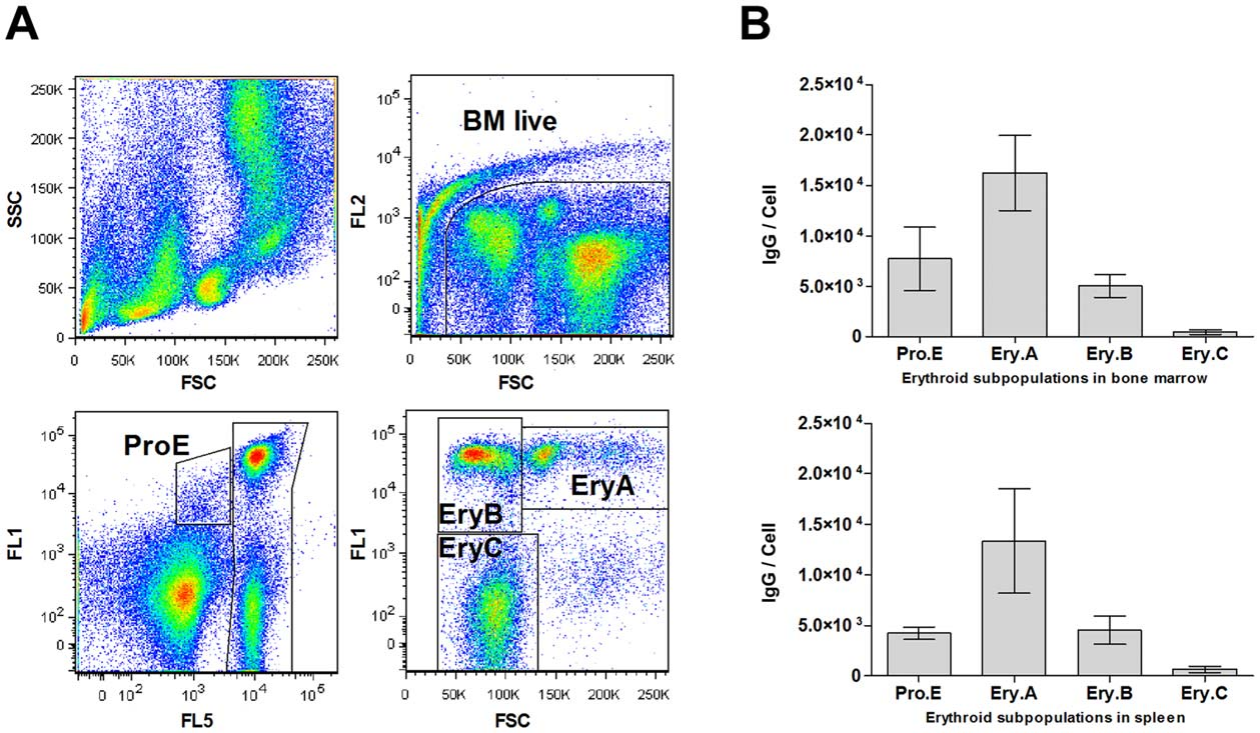
The expression of PrP^C^ on mouse bone marrow (BM) and spleen erythroid precursors is upregulated in early erythroblasts and, then, decreases with the cells' maturation. (A) Gating strategy for erythroid precursors: upper left - scattergram of BM cells; upper right – gating of viable 7-AAD negative cells (BM live); lower left – live BM cells labeled with CD71-FITC (FL1) and Ter119-eFluor450 (FL5): ProE - CD71^+^Ter119^+/−^ proerythroblasts; Ter119^+^ cells were further gated on CD71-FITC (FL1) and FSC plot (lower right): EryA - large CD71^+^ early basophilic erythroblasts, EryB - small CD71^+^ late basophilic and polychromatic erythroblasts, EryC - small CD71^-^ orthochromatic erythroblasts and reticulocytes. (B) Quantitative FACS analysis of PrP^C^ expression on erythroid precursors in mouse bone marrow and spleen. Expression of PrP^C^ on early basophilic erythroblasts (EryA) is significantly higher (p>0.005, n = 5) in comparison with proerythroblasts (ProE), both in BM (upper) and spleen (lower). The initial increase of expression is followed by its decrease on late basophilic and polychromatic erythroblasts (EryB), and most mature small precursors (EryC) express a low number of PrP^C^. Quantification is based on assumption that one IgG molecule of MAb AH6 binds to one molecule of PrP^C^.

Erythroid precursors were gated according their Ter119 and CD71 signals and the forward scatter (FSC) signals to the proE, EryA, EryB and EryC subpopulations (Fig. 1 A). CD71^+^Ter119^+/−^ bone marrow proerythroblasts (proE) expressed 7800±3100 PrP^C^ molecules / cell, assuming that one molecule of mAb AH6 binds one molecule of PrP^C^. The expression of CD71^+^Ter119^+^ basophilic erythroblasts (EryA) was elevated to 16200±3700 PrP^C^ / cell and decreased in late basophilic and polychromatic erythroblasts (EryB) to 5100±1100 PrP^C^ / cell and was also diminished in late CD71^−^Ter119^+^ small precursors (EryC) (470±230 PrP^C^ / cell). Corresponding erythroid precursors in the spleen expressed 4200±600, 13400±5200, 4600±1400 and 680±280 PrP^C^ / cell, respectively (Fig. 1 B).

### Regulation of PrP^C^ expression during the erythroid differentiation of MEL cells

**Figure 2:**
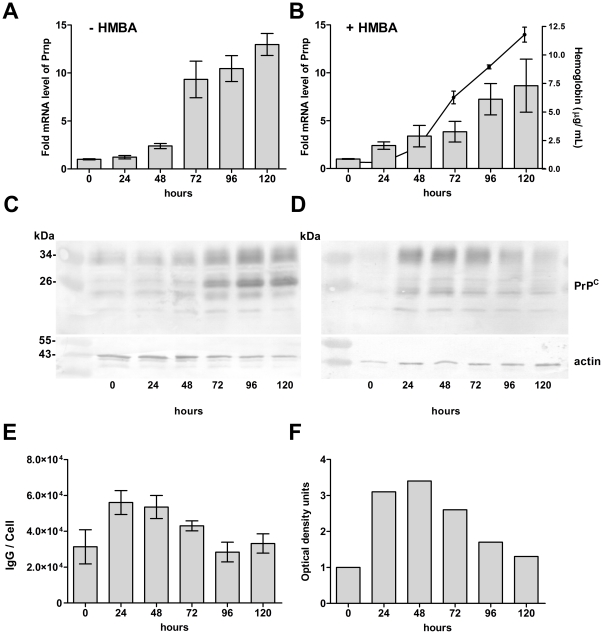
Initial increase of PrP^C^ protein expression in differentiating MEL cells is followed by its downregulation. (A) Transcriptional activation of the Prnp gene in uninduced MEL cells (- HMBA) correlates with growth arrest in confluent culture at 72 h, as demonstrated by qRT-PCR. A similar, but lower, increase of PrP mRNA is seen in cells induced to erythroid differentiation (+ HMBA) (B). Progress of cell differentiation is monitored by overall hemoglobin level (full line, n = 2, mean ± SD). The amount of PrP^C^ protein on western blots (WB) in uninduced cells (C) correlates with the level of mRNA (A). In contrast, the amount of PrP^C^ protein in differentiating cells (D) is highest 24–48 h after induction and, then, is downregulated, as shown by densitometry (F), despite the level of PrP mRNA continues to rise (B). Blots were developed with a mix of PrP^C^ mAbs (AH6, AG4, 6H4) and actin antibody was used as a loading control. Surface expression of PrP^C^ on HMBA induced differentiating MEL cells (number of mAb AH6 IgG molecules / cell) measured by quantitative flow cytometry (E) correlates with the levels detected by WB (F) (n = 2, means ± range).

MEL cells were grown for five days in the absence or presence of 5 mM HMBA. The cells increased their expression of Prnp mRNA, reaching 13±1.2 -fold and 8.7±2.8-fold relative expression after 120 hours in uninduced and differentiating cells, respectively (Fig. 2 A and B). While the level of PrP mRNA in the differentiating cells more than doubled within 24 h after induction, a similar increase in the uninduced cells was observed after 48 h in culture when cells were reaching confluency. At the protein level, the uninduced cells increased their PrP^C^, reaching a maximum expression in confluent culture at 96–120 h (Fig. 2C) which correlated with the expression of Prnp mRNA. In contrast, the expression of the PrP^C^ protein in differentiating cells peaked at 24–48 h post-induction (Fig. 2D) with a subsequent decrease to almost its basal level at 120 h, as demonstrated by densitometry (Fig. 2F). The increased density of the PrP^C^ band on the WB was already visible within 6 h post-induction (not shown). These results were confirmed by quantitative FACS analysis, which demonstrated approximately twofold increase of PrP^C^ membrane expression after 24 h of differentiation, with a subsequent return to the basal level after 96 h (Fig. 2E).

### Dexamethasone treatment of MEL cells lowers their expression of PrP^C^ in confluent culture irrespective of production of hemoglobin

**Figure 3:**
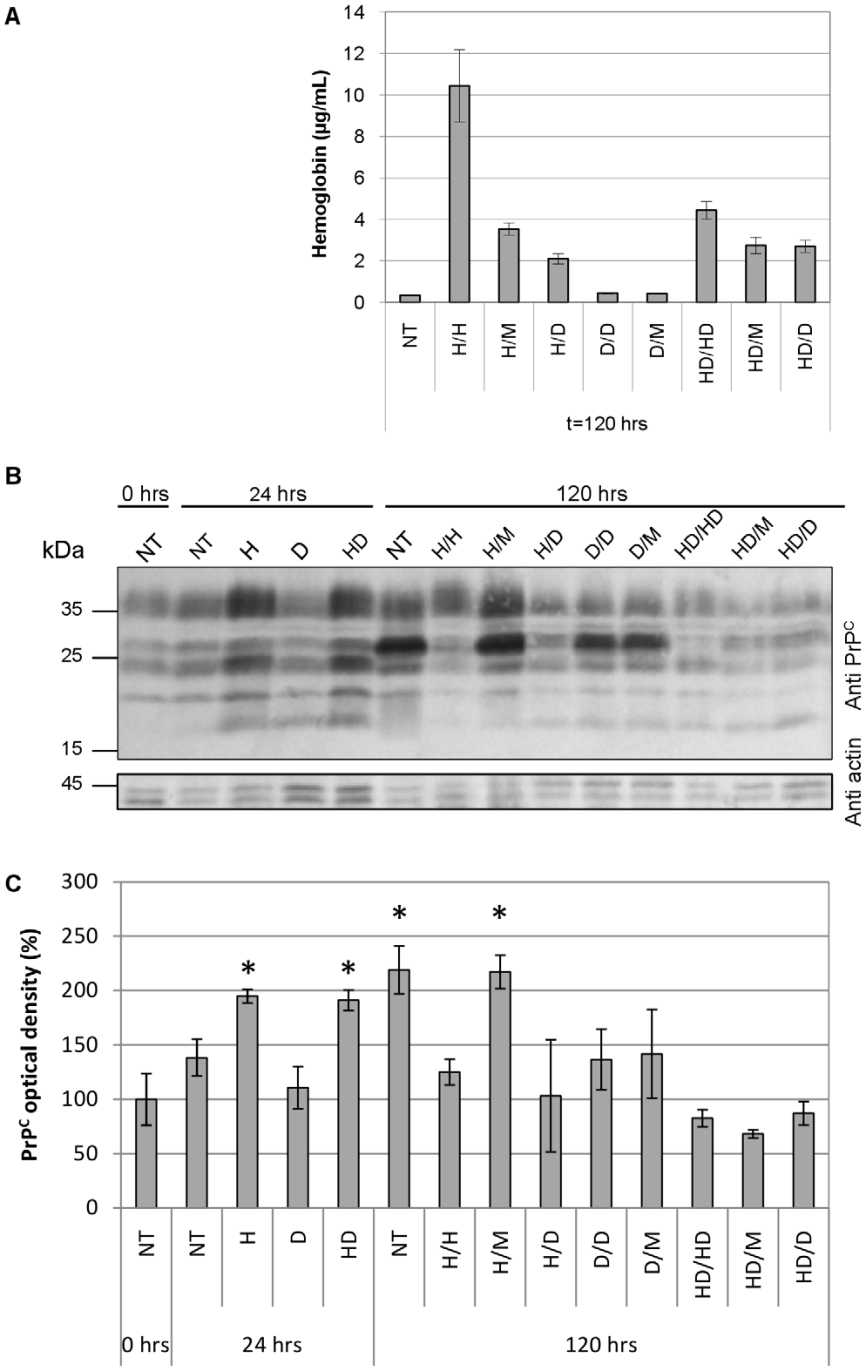
The initial upregulation of PrP^C^ expression after the induction of MEL cells' differentiation by HMBA is not affected by dexamethasone. (A) Hemoglobin levels 120 hrs post induction of MEL cells differentiation. Cells were treated with HMBA (H), Dexamethasone (D) or the combination thereof (HD). After 24 hrs media were exchanged and the treatment continued, or was changed for different one as shown on x-axis labels (M stands for DMEM medium only). Non-treated (NT) control represents cells kept in DMEM only. Cell lysates were normalized to the total protein content (measured by BCA assay) and hemoglobin was quantified by TMB assay. (B) Western blot analysis of PrP^C^ expression. Samples were harvested after 24 and 120 hrs treatments described above. NT control at 0 hrs represents the initial conditions of MEL culture before treatment. The gel loading was normalized to the total protein content (measured by BCA assay) and controlled by actin staining. The blot is a representative of three independent experiments. (C) Quantification of the PrP^C^ level on western blots by densitometry. Graph columns represent the average density of PrP^C^ bands and error bars represent the standard deviations; PrP^C^ expression level of NT control at 0 hrs was set as 100% (*p<0.05, n = 3).

Induction of MEL cell differentiation by incubation with HMBA for 24 h with their subsequent incubation in the media without HMBA (H/M), or the inclusion of 4 µM dexamethasone in the medium with HMBA (HD/HD), both led to a comparable (∼60%) reduction of MEL cells hemoglobinization after 120 h of culture (Fig. 3 A). The H/M treatment was connected with the significant increase of PrP^C^ level (∼220%) at 120 h comparable to the increase recorded in non-differentiated confluent cells (NT) (Fig. 3 B, C). At the other hand, HD/HD treatment led to low PrP^C^ level (∼80%), lower than in differentiated fully hemoglobinized MEL cells (H/H). Treatment of MEL cells with up to 40 µM dexamethasone did not influence the initial up-regulation of PrP^C^ in HMBA stimulated cells 24 h post induction, but generally led to lower PrP^C^ levels in comparison with NT cells after 120 h in culture (Fig. 3 B, C). Interestingly, induction of PrP^C^ expression in growth-arrested confluent culture led to a different PrP western blot pattern with predominant ∼26 kDa band which is much less present in both, fully differentiated or actively dividing MEL cells (Fig. 3 B). The detected changes in the levels of PrP^C^ were not caused by differences in gel loading, as demonstrated by actin labeling.

### Retroviral vector delivery of anti-Prnp shRNAmir leads to stable and efficient PrP^C^ silencing

**Figure 4:**
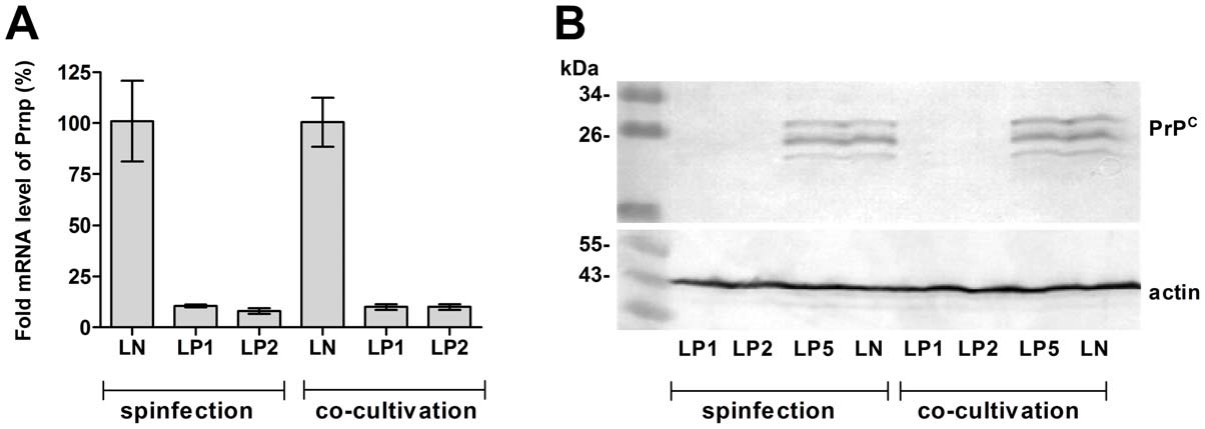
Downregulation of PrP^C^ expression by RNAi in MEL cells after selection with puromycin. (**A**) Both methods of retroviral vector delivery led to ∼90% silencing of Prnp mRNA in both lines expressing anti-Prnp shRNAmir (LP1 and LP2) when compared with the MEL line expressing nonsilencing shRNAmir (LN), as depicted by qRT-PCR. (**B**) Confirmation of PrP^C^ downregulation in LP1- and LP2-transduced cells at the protein level by western blot. PrP^C^ was detected by mAb AH6. No silencing was observed in LP5 cell line. Actin was used as a loading control.

**Figure 5:**
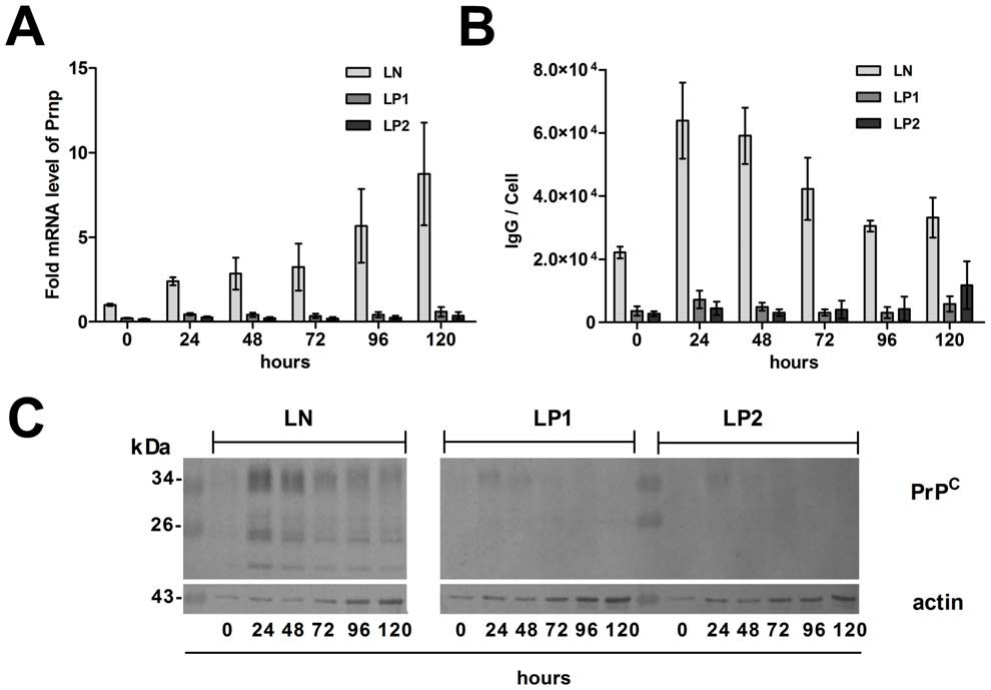
Expression of PrP^C^ is stably repressed during the differentiation of MEL cells. (**A**) Level of PrP mRNA measured with qRT-PCR in cell lines stably expressing anti-Prnp shRNAmir (LP1 and LP2) is downregulated in comparison with control (LN) during the course of cell differentiation (2 experiments, mean ± SD). (**B**) The effect of silencing on the level of PrP^C^ on cell membrane estimated by quantitative flow cytometry (four experiments, mean ± SD. (**C**) Confirmation of stable PrP^C^ silencing on the protein level by western blot. PrP^C^ was detected by mAb mix AH6, AG4 and 6H4. Actin was used as a loading control.

Both spinfection and co-cultivation methods led to efficient (∼90%) silencing of PrP^C^ at the mRNA (Fig. 4 A) and protein levels (Fig. 4 B) by LP1 and LP2 constructs in comparison with control construct LN. The LP5 construct did not produce any downregulation of PrP^C^ levels (Fig. 4 B). Retroviral infection of cells did not induce cell-protective effects, as demonstrated by qRT-PCR analysis of chosen interferon-stimulating genes *Oas1a*, *Rnase1* and *Eif2ak2* (not shown). The silencing of PrP^C^ in LP1 and LP2 lines was stable during the course of HMBA-induced cell differentiation (Fig 5). In comparison with LN, LP1- and LP2-transduced cells exhibited 79% and 84% inhibition of Prnp mRNA expression at the beginning of differentiation and 93% and 96% after 120 h of differentiation, respectively (Fig. 5 A). The levels of Prnp mRNA and protein during differentiation of the control LN-transduced cells (Fig. 5 A, C) was upregulated similarly as in non-manipulated MEL cells (Fig. 2 B, D). The stability of PrP^C^ silencing was confirmed by quantitative FACS analysis, which demonstrated downregulation of PrP^C^ at the cell membrane ranging from 17% (LP1) and 12% (LP2) to 8% (LP1) and 5% (LP2) of its expression (100%) in LN-transduced cells at the beginning of differentiation and after 48 h, respectively (Fig. 5 B). These results were confirmed by western blot analysis in which we could faintly detect PrP^C^ in LP1 and LP2 cells only after 24 hours, while the protein in LN-transduced cells was readily detected (Fig. 5 C).

### Silencing of the Prnp gene by RNAi does not affect HMBA-induced differentiation of MEL cells

**Figure 6:**
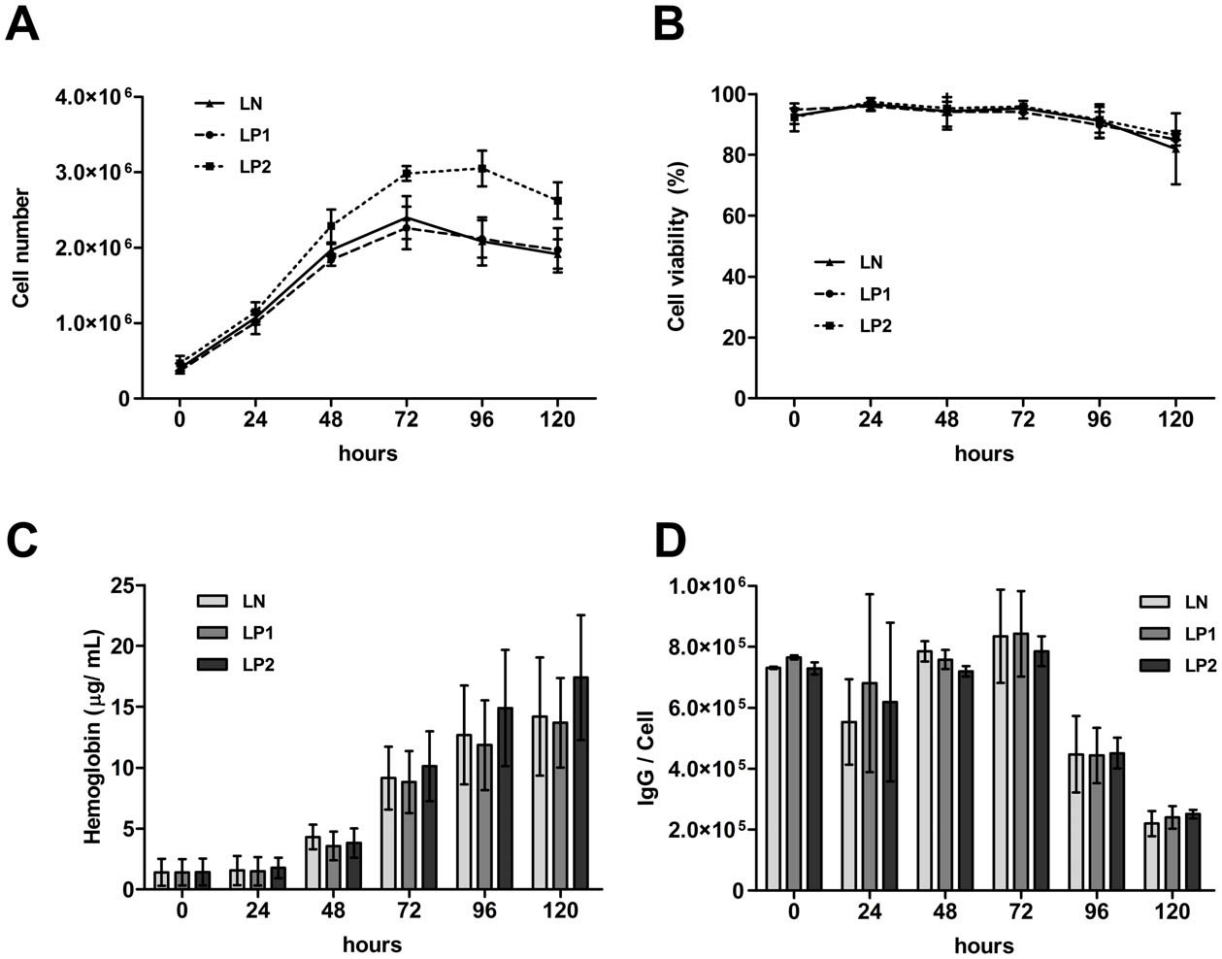
Differentiating MEL cells display similar characteristics irrespective to the level of PrP^C^ expression. (**A**) Growth curves of LP1, LP2 and control LN lines during erythroid differentiation induced by HMBA. (**B**) Cell viability based on a Trypan Blue exclusion assay. (**C**) Concentration of total hemoglobin in the cells per 100 µg/mL of total cell proteins. (**D**) Number of CD71 (transferrin receptor) molecules per cell, analyzed by quantitative flow cytometry, based on assumption that one anti- CD71-PE mAb binds one molecule of transferrin receptor.

**Figure 7:**
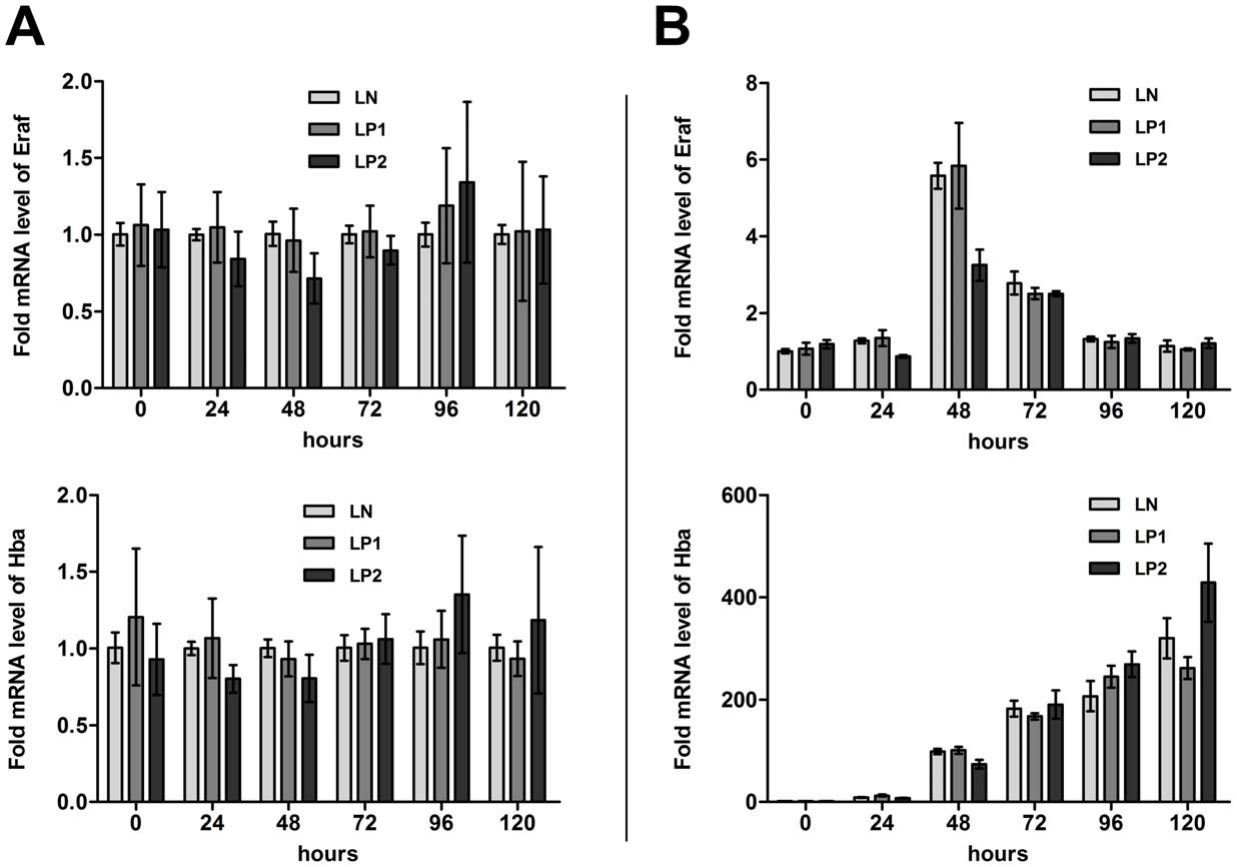
Transcriptional levels of selected erythroid markers in differentiating MEL cells indicate a similar pattern, irrespective to PrP silencing. (A) Expression of Eraf (α-hemoglobin stabilizing protein) and Hba (Hemoglobin α) in LP1- and LP2-transduced cells was compared with the level in the control LN-transduced cell line on individual days, as measured by qRT-PCR (n = 4, mean ± SD). (B) Representative pattern of Eraf and Hba expression during differentiation. The level of expression in individual days was normalized to the level in the LN-transduced cell line in day 0.

Cell numbers in differentiating LN and LP1 lines increased similarly, reaching 2.4×10^6^ cells/mL at 72 h. The LP2-transduced cell line produced slightly more cells, with a maximum of 3.0×10^6^ cells/mL at 72 h (Fig. 6 A). In a Trypan Blue exclusion assay, all cell lines demonstrated a similar viability (∼94%) over a 72-h post-induction period and, also, a similar slight decrease of viability after 96 and 120 h (Fig. 6 B). We did not detect any differences in the transcriptional levels of proapoptotic Bax (not shown). The dynamic of hemoglobin production was comparable in all cell lines, irrespective of the level of PrP^C^ (Fig. 6 C). Similarly, surface expression of the transferrin receptor (CD71) was similarly regulated in all cell lines (Fig. 6 D). Also, no difference was demonstrated in the levels of c-myb mRNA, which were downregulated approximately eightfold after 24 h post-induction in all lines. After 48 h, c-myb mRNA was downregulated to ∼2% of the starting level and remained silenced until the end of the experiment (not shown). Similarly, monitoring of the erythroid markers Eraf and Hba (Fig. 7A and B) and GATA-1 (not shown) during the differentiation of cells at the transcriptional level indicated analogous expression in all lines, irrespective of PrP^C^ levels.

## Discussion

The role of PrP^C^ in cellular physiology has been proposed for a variety of processes, but with no prevailing consensus to date[1]. The downregulation of erythroid genes during prion infection has established a link between the peripheral pathogenesis of prion diseases and erythropoiesis. However, it is not clear if the effect is caused by direct interaction of prion particles with erythroid cells or if it is triggered by some yet unknown humoral response to the infection. The expression of PrP^C^ on circulating red blood cells (RBCs) of closely related nonhuman primates varies from several thousand per cell to zero[15], implying that its function on mature erythrocytes is not conserved. Griffiths et al. demonstrated the regulation of PrP^C^ expression during the differentiation of cultured human erythroblasts *in vitro*, indicating that it may play a role in the differentiation of erythroid precursors. PrP^C^ was found mostly in the perinuclear region of proerythroblasts, and the amount of PrP^C^ declined as erythroid cells matured[16]. Human and mouse RBCs express approximately 200 PrP^C^ molecules per cell[11], suggesting a similar regulation of its expression in the erythroid lineage in both species. In this study, we demonstrated that the surface expression of PrP^C^ on erythroid precursors in the mouse bone marrow and spleen follows a similar pattern as the cells mature. The protein's levels first increase with basophilic erythroblasts, expressing more than twice as much PrP^C^ as proerythroblasts, and then declines significantly in late basophilic and polychromatic erythroblasts, with most mature small erythroid precursors expressing only around 500 PrP^C^ molecules per cell. This pattern of expression is suggestive of PrP^C^'s involvement in the early stages of erythroid differentiation. The differentiation of MEL cells in culture is known to resemble the *in vivo* process, with similar maturation stages: proerythroblast-like, erythroblasts-like and, occasionally, reticulocyte-like[16], [17]. The surface expression of PrP^C^ on uninduced MEL cells in the exponential phase of growth was approximately six times higher than that observed on proerythroblasts in the mouse bone marrow or spleen. However, the induction of differentiation led to nearly doubling of the PrP^C^ number on MEL cell membranes within 24 h, which resembled a similar increase of PrP^C^ expression on basophilic erythroblasts *in vivo*. In agreement with previous observations *in vivo*, we found that this initial upregulation was followed by a gradual downregulation of PrP^C^ surface levels along with the progression of MEL cells' differentiation. This decrease did not reach the degree observed in small CD71^-^Ter119^+^ erythroid precursors *in vivo*, which is in line with the limited ability of differentiating MEL cells to reach this stage of maturation. The differentiation of MEL cells is composed of two separate events, growth arrest and terminal differentiation. Both processes are characterized by the activation of early (cell-cycle control) and late (morphological changes) genes[17]. The first period lasts approximately 12 hours, after which the first committed cells materialize. The majority of cells are terminally differentiated between 24 and 50 h[18]. In agreement with previous studies by Gougomas et al.[14], we found that the Prnp gene in MEL cells is transcriptionally activated both during inducer-mediated differentiation and in confluent cells undergoing cell-cycle arrest. In addition, we were able to demonstrate the regulation of PrP^C^ at the protein level by western blot. Interestingly and in accordance with the flow cytometry results, in differentiating cells, the expression of the protein was downregulated after an initial increase, even though the level of PrP mRNA continued to rise. This result suggests that MEL cells' differentiation leads to a translational regulation of PrP^C^ levels[19] not seen in uninduced cells undergoing cell-cycle arrest. Alternatively, more differentiated cells could degrade PrP^C^ at an increased rate, as has been proposed to explain the disparity between PrP protein and mRNA levels in different types of neuronal cells[20]. The downregulation of PrP^C^ protein during the differentiation of highly responsive subclones of MEL cells was recently reported by Otsuka et al.[21]; however, their study did not detect an initial increase of PrP^C^ expression, most likely due to the different status of cells at the point of induction. Stimulation of the glucocorticoid receptor by dexamethasone induces the proliferation and expansion of erythroid progenitors and delays the terminal differentiation of erythrocytes[22]. In our hands, dexamethasone did not prevent the HMBA-induced initial upregulation of PrP^C^ in MEL cells, suggesting that it precedes the effect of dexamethasone, which is known to suppress the HMBA-mediated commitment to terminal cell division at a relatively late step in this process[23]. However, dexamethasone prevented the increase of PrP^C^ protein levels in confluent MEL cells after 120 h of culture, demonstrating that the activation of the glucocorticoid receptor can interfere with the transcriptional activation of the Prnp gene mediated by cell-cycle arrest. The mechanism of dexamethasone's action on the prevention PrP^C^ protein upregulation in confluent MEL cells is unknown at present. Dexamethasone has been shown to induce cell-cycle arrest in number of various cell lines[24], but not in MEL cells, in which it increases cell viability[25], both in induced and uninduced culture. In summary, our results demonstrate that the regulation of PrP^C^ levels in differentiating MEL cells resembles, at least in part, its regulation in maturing mouse erythroid precursors *in vivo*. To learn more about the importance of PrP^C^ in the process of MEL cells' differentiation, we created cell lines using RNAi to stably inhibit expression of the protein. RNAi administered by shRNA from a retrovector had previously been employed efficiently to inhibit PrP^C^ expression *in vitro* and *in vivo* [26], [27]. The main objective for using RNAi to suppress PrP^C^ was to study its therapeutic potential in preventing propagation of infectious prions. To the best of our knowledge, our model is the first murine cell line of non-neuronal origin with stably silenced PrP^C^ expression. Inhibition of the protein's expression at both the mRNA and protein levels was efficiently maintained during the differentiation of MEL cells, although it varied between 75 and 95% in individual time points. Despite the silencing, the induction of differentiation led to a detectable increase of PrP^C^ signal on blots after 24 h, suggesting that the regulation of the protein's expression in LP1- and LP2-transduced cell lines follows similar pattern as in unmodified MEL cells, although at a suppressed level. Growth curve and viability of LP1-, LP2- and control LN-transduced cell lines after the induction of differentiation was similar, although the LP2-transduced cell line exhibited a higher proliferation capacity. Since the LP1-transduced cell line did not differ from control LN-transduced cell line, we could not assign the LP2-transduced cell line's divergence solely to PrP^C^ silencing. All cell lines observed here demonstrated similar dynamics and level of hemoglobinization and regulation of the transferrin receptor on their cell membranes. This finding suggested that silencing of PrP^C^ in MEL cells does not lead to gross perturbation of iron homeostasis, although the involvement of PrP^C^ in iron-cell uptake was described recently[28], [29]. Similarly, the level of the protooncogene c-myb, expression of which is characteristic of the proliferative state and has been demonstrated to block MEL cells' differentiation[30], [31], decreased upon induction similarly in all created cell lines and remained low during the entire course of the experiment. Monitoring of selected erythroid markers (AHSP, Hba and GATA-1) on the transcriptional level also did not reveal significant differences among LP1-transduced, LP2-transduced and LN-transduced cell lines, confirmingthat PrP^C^ silencing does not appear disturb the differentiation of MEL cells. In many cell cultures, the enhanced expression of PrP^C^ was proposed to facilitate cytoprotective effects[32]. However, overexpression of exogenously delivered PrP^C^ in MEL cells did not protect the cells against apoptosis initiated by serum withdrawal[33]. We also found that silencing of PrP^C^ did not seem to sensitize cells to apoptosis during differentiation, as demonstrated by a Trypan Blue exclusion assay and by monitoring of Bax expression by qRT-PCR. This result is concurrent with Christensen and Harris, who reevaluated former assays reporting a protective activity of PrP^C^ and suggested that the presence of PrP^C^ has only a modest effect in cytoprotection *in vitro* [34].

Taken together, our results imply that, under normal conditions, PrP^C^ seems dispensable for the erythroid differentiation of MEL cells. The pattern of PrP^C^ regulation on erythroid precursors *in vivo* suggests that PrP^C^ may play a role in the maturation of erythroblasts in erythroblastic islands. We can speculate that PrP^C^, which was shown to bind both laminin and the laminin receptor[35], [36], can be involved in cell-cell contacts in an erythroblastic niche, or with a surrounding extracellular matrix. Downregulation of PrP^C^, then, could play a role in the dissociation of matured reticulocytes from the erythroblastic niche. Such a role for PrP^C^ is unlikely to be detected by the MEL cell model. Another explanation could be that PrP^C^ exerts its function only under stress conditions. This hypothesis is consistent with the documented poor recovery of PrP^−/−^ mice from experimental anemia[7]. Finally, it is possible that the effect of PrP^C^ silencing was compensated for by an unknown pathway or that the remaining expression is sufficient to sustain its role.

In conclusion, we created model cell lines with efficiently silenced expression of PrP^C^, which are the first of their kind and may serve as a valuable tool in subsequent searches for the function of PrP^C^ beyond the erythroid differentiation.

## Materials and Methods

### Flow cytometry of mouse bone marrow and spleen erythroid precursors

The study was approved by the Committee on the Ethics of Animal Experiments of the First Faculty of Medicine, Charles University in Prague (Permit Number: 217/07). Bone marrow (BM) cells were isolated from the femurs of PrP^+/+^ mice (C57BL/6×129/SvxCD1 mixed background) by washing with PBS, 1% BSA and 2 mM EDTA, pH 7.4 (PBS-BE). Spleen and BM cells were passed through a 40-µm cell strainer (Becton Dickinson, San Diego, CA, USA) to eliminate stroma and debris. The cells were labeled (30 min, 4°C) with saturating concentration of monoclonal antibodies (mAbs): anti-mouse Ter-119 eFluor 450, anti-mouse CD71-FITC (both eBioscience, San Diego, CA, USA) and AH6 (TSE Resource Center, Roslin, UK), custom conjugated to PE (Exbio, Vestec, Czech Republic). Labeled cells were washed and resuspended in PBS-BE, and their fluorescence was analyzed on a BD FACSCanto II flow cytometer equipped with BD FACSDiva Software v6.0 (Becton Dickinson). Dead cells were excluded from the analysis by 7-aminoactinomycin D (7-AAD) (Molecular Probes, Eugene, OR, USA) labeling, and the fluorescence of live cells (10^5^) was analyzed. Erythroblast subpopulations were resolved according Ter119, CD71 and forward-scatter (FSC) signals. Proerythroblasts (proE) were identified as Ter119^medium^ CD71^+^ cells. Ter119^high^ cells were divided using both the CD71 and FSC parameters into three populations, labeled EryA, EryB and EryC, as described previously[37], and the binding of the prion mAb AH6 was quantified using Standard Quantum R-PE MESF beads (Bangs Laboratories, Fishers, IN, USA)[11], [15].

### Flow cytometry of cell cultures

Cells were washed with 1% BSA-PBS, pH 7.4 (PBS-B) and labeled for 20 min at RT with saturating concentrations of the AH6-PE or anti-mouse CD71-PE (eBioscience) mAbs. Next, washed cells were resuspended in PBS-B and analyzed using a flow cytometer to standardize the fluorescence readings and to allow for the quantification of PrP^C^ and CD71 expression Standard Quantum R-PE MESF beads were run as a separate sample. Viable cells were identified as a 7-AAD-negative population.

### Cell cultures

The MEL cell line (clone 707, ECACC, Salisbury, UK) and packaging cell line HEK293 GP2 (Clontech, Mountain View, CA USA) were maintained in DMEM growth medium containing high glucose (4.5 g/L), L- glutamine, sodium pyruvate, 10% fetal bovine serum and puromycin/streptamycin (all reagents from PAA, Pasching, Austria). Cells were cultured at 37°C in a humidified atmosphere containing 5% CO_2_. For all experiments, MEL cells between the second and fourth passages were seeded at a density of 10^5^ cells/mL 24 h before induction of differentiation by 5 mM HMBA (final concentration). In some instances, 4 µM dexamethasone (Sigma-Aldrich, Prague, Czech Republic) was used to block the commitment of MEL cells. The cells were incubated with either HMBA, HMBA and dexamethasone or dexamethasone alone for 24 h, and then the medium was changed, and the cells were grown in the presence of either HMBA, dexamethasone, HMBA and dexamethasone, or in medium only.

### Cloning

For the silencing of cellular prion expression, we used shRNAmir sequences HP_285770 (LP1) and HP_288208 (LP2), which are available in the RNAi codex database (http://cancan.cshl.edu/cgi-bin/Codex/Codex.cgi). The shRNAs, together with the control nonsilencing shRNAmir (LN), were ordered in the pSM2 retro vectors V2MM_66187, V2MM_63696 and RHS1707 (Open Biosystems, Huntsville, AL, USA)[38]. The third anti- Prnp mRNA sequence (LP5), adopted from Pfeifer et al., was purchased as an oligonucleotide[27]. All sequences were cloned into the LMP retrovector MSCV/LTRmiR30-PIG (Open Biosystems). Constructs created in this study were verified by sequencing.

### RNAi of Prnp expression

Packaging HEK293 GP2 cells were seeded at a density of approximately 1.5×10^5^ cells/well (24-well plate). After 24 h, the cells were transfected with a mixture of the VSV-G plasmid, coding the envelope protein of the vesicular stomatitis virus (Clontech), and the appropriate LMP retrovector (LN, LP1, LP2 or LP5). Plasmids were delivered in a ratio of 9:1 with the Arrest-in transfection agent (Open Biosystems). Three days after transfection, media containing retroviral particles were centrifuged (400 g, 4°C) to pellet eventual cell contaminants, and the infectious supernatant, containing 4 µg/mL Polybrene (Sigma-Aldrich), was added to MEL cells in a 1:1 ratio. Subsequently, transduced MEL cells were spun down at 450 g for 90 min at RT (a method known as spinfection). In a second approach, the MEL cells were infected by co-cultivation with a packaging cell line. In this method, medium in HEK293 GP2 cells was aspirated 24 h postinfection, and 2×10^4^ MEL cells/well were added inside the 0.4-µm-pore translucent polyphthalate insert (Becton Dickinson) in a 0.7 mL total volume of fresh media containing 4 µg/mL Polybrene. After 48 (co-cultivation) or after 72 (spinfection) hours of incubation, the cells were diluted with fresh medium, and selection started the next day through the addition of puromycin (Sigma) at a final concentration of 0.5 µg/mL. The percentage of cells with an integrated retrovector genome was monitored by FACS utilizing eGFP positivity. Cell lines prepared by both methods were mixed after three weeks after reaching >95% eGFP positivity and were frozen in aliquots.

### Western blot and densitometry

Cells were washed in PBS and lysed with 1% TX-100, 0.5% sodium deoxycholate and 0.1% SDS in 150 mM NaCl, 2 mM MgCl_2_ and 50 mM Tris, pH 8.0, supplemented with 12 units/µL benzonase (Sigma) and the EDTA-free protease inhibitor Complete (Roche Diagnostics, Basel, Switzerland). Protein concentration was measured by BCA assay (Thermo Fisher Scientific, Rockford, IL, USA), and 20 µg proteins/lane were loaded for SDS-PAGE analysis. Separated proteins were blotted to nitrocellulose membranes (Bio-Rad, Hercules, CA, USA) and PrP^C^ detected with a mix of the mAbs AH6, AG4 (both 1 µg/mL, TSE resource center) and 6H4 (0.05 µg/mL, Prionics AG, Zurich, Switzerland). The anti-actin polyclonal Ab I-19 (Santa Cruz, Santa Cruz, CA, USA) (0.5 µg/mL) was used as a loading control. Secondary antibodies were goat anti mouse IgG F(ab)_2_ alkaline phosphatase (Biosource) and goat anti rabbit IgG F(ab)_2_ alkaline phosphatase (Caltag, Buckhingham, UK), both at a dilution of 1: 4000. BCIP/NBT (Caltag) was used as a chromogen. The density of bands on blots was quantified using MiniLumi densitometer software (DNR Bio-Imaging Systems Ltd., Israel).

### Reverse transcription and quantitative PCR

RNA was isolated using the RNA Blue solution according to the manufacturer's manual (Top-Bio, Prague, Czech Republic). Contaminating genomic DNA was degraded by treatment with TURBO DNase (Ambion, Austin, TX, USA). RNA integrity was evaluated by electrophoresis, and 0.5 µg RNA was reverse-transcribed with the RevertAid first-strand cDNA synthesis kit (Fermentas, Burlington, ON, Canada) according to the manufacturer's manual. Complementary DNA was tenfold diluted, and 2 µL were used for qRT-PCR performed in an ABI 7300 PCR System using TaqMan primers, probes and the Universal PCR Master Mix (Applied Biosystems, Carlsbad, CA, USA). We monitored the expression of the following genes: glyceraldehyde-3-phosphate dehydrogenase (*Gapdh*), the prion protein (*Prnp*), the hemoglobin alpha adult chain 1 (*Hba-a1*), the myeloblastosis oncogene (*Myb*), the α-hemoglobin stabilizing protein (*Eraf*), the GATA-binding protein 1 (*GATA-1*), 2′-5′ oligoadenylate synthetase 1A (*Oas1a*), ribonuclease L (*Rnase1*), the eukaryotic translation initiation factor 2-alpha kinase 2 (*Eif2ak2*) and the BCL2-associated X protein (*Bax*). Relative expression levels were calculated using the 2^-ΔΔCT^ method[39] and normalized to the reference Gapdh gene. Expression quantities were normalized as described in results.

### Spectrophotometric determination of hemoglobin content

The hemoglobin concentration in MEL cell lysates was measured with the TMB assay as described previously, with adjustments for 96-well plates[40]. The cell lysates were diluted to contain 100 µg of protein per mL, and their absorbance at 660 nm was measured using a VICTOR^2^ D fluorometer (PerkinElmer, Waltham, Massachusetts, USA). The amount of hemoglobin was subtracted from the calibration curve composed of serial dilutions of purified hemoglobin (Sigma).
